# Head-to-Head Comparison of Anti-Inflammatory Performance of Known Natural Products *In Vitro*

**DOI:** 10.1371/journal.pone.0155325

**Published:** 2016-05-10

**Authors:** Iris E. Allijn, Stefan F. C. Vaessen, Linda C. Quarles van Ufford, Kees J. Beukelman, Menno P. J. de Winther, Gert Storm, Raymond M. Schiffelers

**Affiliations:** 1 Department of Biomaterials Science and Technology, University of Twente, Enschede, The Netherlands; 2 Technology & Innovation, Innovative testing in Life Sciences and Chemistry, University of Applied Sciences Utrecht, Utrecht, The Netherlands; 3 Medicinal Chemistry & Chemical Biology – Biomolecular Analysis, Department of Pharmaceutical Sciences, Utrecht University, Utrecht, The Netherlands; 4 PhytoGeniX BV, Bunnik, The Netherlands; 5 Department of Medical Biochemistry, Academic Medical Center, Amsterdam, The Netherlands; 6 Department of Pharmaceutics, Utrecht University, Utrecht, The Netherlands; 7 Clinical Chemistry and Haematology, University Medical Centre, Utrecht, The Netherlands; Queen Mary University of London, UNITED KINGDOM

## Abstract

Inflammation is an important therapeutic target. Due to their potency, steroidal drugs dominate the current treatment of inflammatory disorders. However, steroidal drugs can also exert a broad range of side effects and appear not always effective. This calls for the development of alternative drugs with a different mechanism of action, which are likely to be found in the field of natural products (NPs). For many NPs strong anti-inflammatory effects have been described, but usually investigating a single compound in a single assay. In this study, eight promising NPs were selected and tested against the strong anti-inflammatory drug prednisolone. For this head-to-head comparison, *in vitro* assays were used which represent different pathways of the inflammatory response: TNF-α and IL-6 expression by macrophages, IL-8 expression by colon epithelial cells, ROS production in polymorphonuclear leukocytes and platelet activation in whole blood. Performance profiles were established which allowed us to identify curcumin, berberine chloride and epigallocatechin gallate as potential alternatives for prednisolone or other glucocorticoids in inflammation.

## Introduction

**Fig 1:**
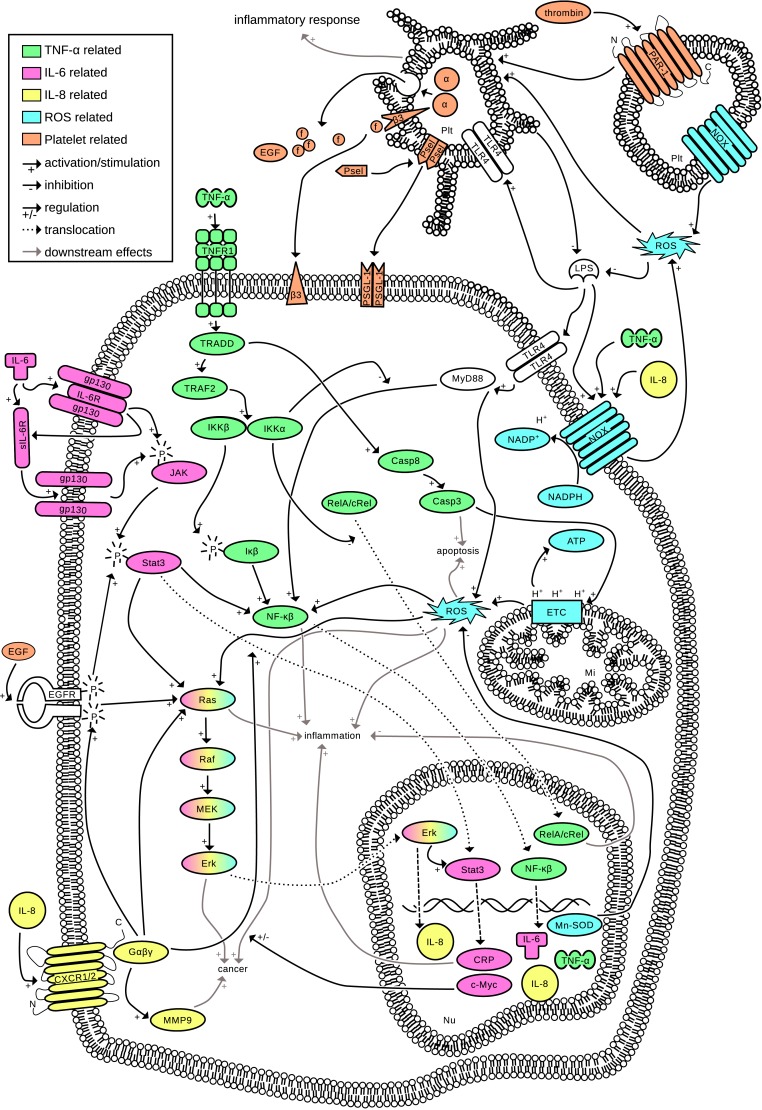
Interconnection of inflammatory pathways. Five important processes in inflammation are combined into one interconnected pathway network. (**TNF-α, green**) TNF-α is part of the very extensive NF-κβ pathway [3]. TNF-α starts multiple signaling cascades by recruiting the tumor necrosis factor receptor 1 (TNFR1), which is subsequently recruiting the TNFR1 associated death domain (TRADD) [4]. TRADD on one side activates the caspase cascade which leads to apoptosis and ROS production [5]. On the other side, the core component complex IKKα/β (Iκα/β kinase) of the NF-κβ pathway is activated. The β part subsequently phosphorylates Iκβ which in turn activates NF-κβ [4]. This leads to translocation of NF-κβ dimers to the nucleus and upregulation of (among others) IL-6, IL-8, TNF-α, and manganese superoxide dismutase (Mn-SOD) [4–6]. (**IL-6, pink**) IL-6 is an important activator of the Janus kinase signal transducer and an activator of transcription [7]. The JAK/Stat pathway is involved in the upregulation of pro-inflammatory cytokines in inflammation, cell proliferation and tumorigenesis [6,8]. IL-6 binds to the IL-6 receptor (IL-6R), which in turn associates with the gp130 protein complex on the cell membrane and phosphorylates JAK. Only a few cell types express the IL-6R on the cell membrane, however, all cells have a soluble form of this receptor (sIL-6R) and the gp130 dimer, meaning that JAK/Stat signaling can be activated in essentially all cell types. The complexation of IL-6 with gp130 and consequently the phosphorylation of Stat3 is needed for a controlled inflammatory response [9,10]. Activated Stat3 dimerizes and translocates to the nucleus, where (among others) c-Myc and c-reactive protein (CRP) are upregulated [10]. Furthermore, activated Stat3 stimulates NF-κβ [2] and the Ras oncogene which is important in both the development of cancer [6,10] and stimulation of inflammation [6]. (**IL-8, yellow**) IL-8 is a pro-inflammatory chemokine whose expression is primarily regulated by NF-κβ. IL-8 binds to G-coupled protein receptor CXCR1/2, which in turn stimulates the Ras oncogene and promotes the nuclear translocation of Stat3 [11]. It is the most powerful human neutrophil chemoattractant and stimulates tumor growth. Furthermore, TNF-α and ROS are potent inducers of IL-8 production [12]. (**ROS, blue**) At an inflammatory site, ROS (which include superoxide radicals, nitric oxide and hydrogen peroxide [5]) are produced continuously (the oxidative burst) as one of the first lines of attack against pathogens [13]. ROS production is vital in acute inflammation, however, a too high production of ROS can cause DNA repair failure [6] and modifications in proteins [13], and are carcinogenic [5]. Intracellularly, most ROS are produced by the mitochondrial electron transport chain (ETC), which is also stimulated in response to TNF-α [5]. These ROS are important for apoptosis as well as cell maintenance, but also stimulate NF-κβ, inflammation and cancer [13]. ROS can also activate platelets [14]. (**Platelets, orange**) Platelets are derived from megakaryocytes, do not have a nucleus and are essential for hemostasis and thrombosis. However, platelets are also loaded with immune modulators, and can drive the inflammatory response. Platelets express NADPH oxidase (NOX) and are an important source of ROS [15]. Upon activation by thrombin or ROS, α-granules are secreted which contain (among others) fibrinogen, P-selectin and EGF (15)⁠. f = fibrinogen, Psel = P-selectin, EGF = endothelial growth factor, α = α-granules, β3 = β-integrin receptors, LPS = lipopolysaccharide, Plt = platelet, Nu = nucleus.

Inflammation is an important process to defend against pathogens and injuries. A controlled acute inflammatory response is beneficial for the body. However, inflammation can become detrimental when the process is dysregulated. Uncontrolled inflammation is underlying most chronic diseases such as cardiovascular disease, arthritis, asthma and type 2 diabetes mellitus [1] and is often linked to cancer development [1,2]. Inflammation is a complex process involving many mediators, with TNF-α, IL-6, IL-8, ROS and platelet activation being key players (Fig 1). Even though Fig 1 addresses only a limited number of pathways, the complex nature of inflammation and the many mediators involved is apparent.

To modulate inflammatory responses, a variety of anti-inflammatory drugs are used, which can be broadly categorized as non-steroidal anti-inflammatory drugs and steroids. Glucocorticoids (GCs) are the most robust anti-inflammatory agents known [16], widely used [17] and the most effective drugs in many chronic inflammatory and immune diseases [18,19]. In general, GCs decrease the transcription of pro-inflammatory cytokines and chemokines and increase the transcription of anti-inflammatory cytokines [17,18]. In addition, there are also non-genomic actions described for GCs [16].

Despite their strong anti-inflammatory activities, a variety of systemic side effects [17] can outweigh the benefits of GC treatment [20]. About 90% of patients with chronic GC treatment develop side effects, ranging from mild (acne) to severe (Cushing Syndrome) and even life threatening events (heart disease) [19]. Besides the adverse effects, individual patients can respond differently to GCs and, with chronic use, many patients develop a form of GC resistance [16]. A small number of patients is even completely resistant to initial GC therapy [18]. The need for alternative treatments is therefore substantial [17,18]. Particularly compounds with a different anti-inflammatory mechanism of action would be attractive. Such compounds might be found in the imminent field of natural products (NPs) because of their described anti-inflammatory bioactivity and great structural variety [21].

NPs show an enormous structural diversity [21,22]. They are important metabolites in plants and play key roles in antibiotic mechanisms, defense against herbivores, protection against UV radiation, nutrition and growth of the plant [23]. NPs are an important source for new drug development [21]. Indeed, half of the drugs in clinical use today is of natural origin [22,24].

In this study, in order to find active but less toxic alternatives for GCs, we have selected eight NPs because of their alleged anti-inflammatory properties and popularity. We have compared their therapeutic *in vitro* efficacy with that of the GC prednisolone (PLP, Fig 2.1). We chose PLP, because it is an often prescribed GC which is with its intermediate potency a good representative of the GCs. As such, it serves as a good benchmark for the potency of the natural products in comparison to a strong anti-inflammatory compound. Although these eight NPs are well known, and their anti-inflammatory activity has been described, the head-to-head comparison in multiple anti-inflammatory assays, usually applied in pharmacology, is novel in phytomedicine.

**Fig 2:**
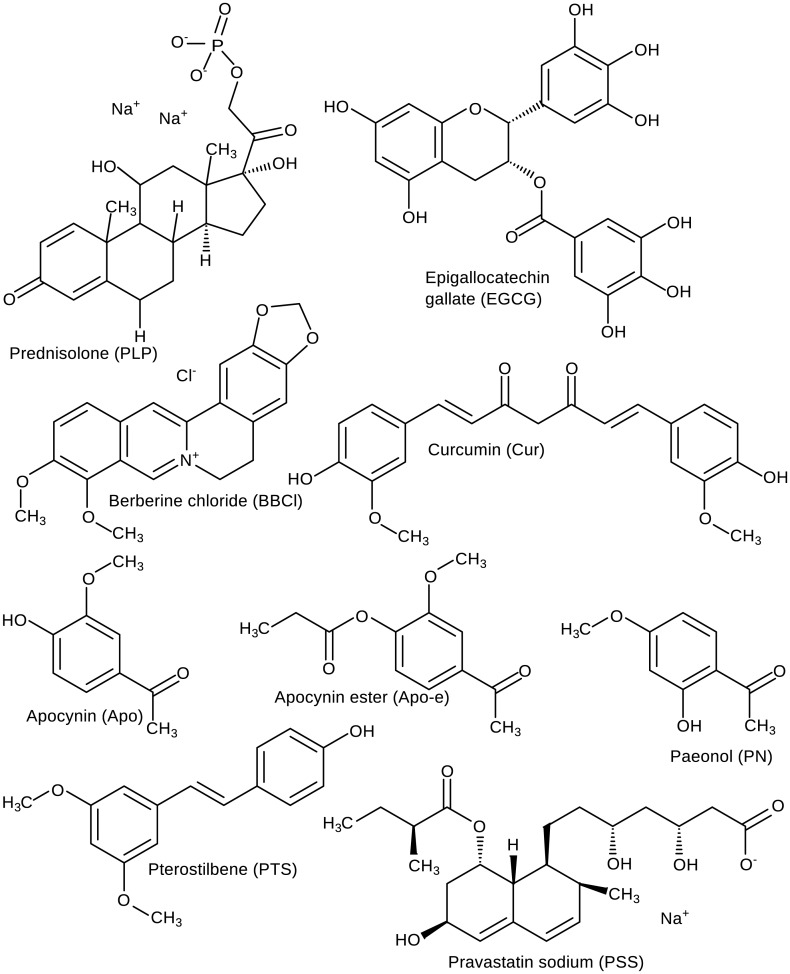
Molecular Structures. The main microspecies at pH 7.4 are depicted for the reference corticosteroid and the selected natural compounds. PLP = prednisolone disodium phosphate, EGCG = epigallocatechin gallate, BBCl = berberine, Cur = curcumin, Apo = apocynin, Apo-e = apocynin ester, PN = paeonol, PTS = pterostilbene, PSS = pravastatin sodium. (drawn using MarvinSketch 6.1.7, ChemAxon [39]).

Epigallocatechin gallate (EGCG, Fig 2.2), the main constituent in green tea leaves (*Camellia sinensis)* [25] has gained a lot of interest in the past decade [26]. It is a good radical scavenger with chemopreventive actions [23] and anti-inflammatory effects [26].

Berberine chloride (BBCl, Fig 2.3) is an alkaloid found in barberry plants (*Berberis species*) [27], which have a long history in traditional medicine [27,28]. BBCl is used in diabetes mellitus against insulin resistance [29]. Pharmacological properties of BBCl include antimicrobial, antidiarrheal [28], anti-inflammatory and anti-oxidant [21,28,29].

Curcumin (Cur, Fig 2.4) is a pigment [23] isolated from *Curcuma longa* which has been used in many ailments in traditional Indian medicine [30]. Cur has chemopreventive actions [23] and, despite its poor bioavailability, is used in the development of new anti-inflammatory and anti-cancer drugs [21].

Apocynin (Apo, Fig 2.5), isolated from *Picrorhiza kurroa* [31], is well known in traditional medicine [32], and inhibits NADPH oxidase [14,32] and platelet recruitment [14]. Apo dimerizes upon entering the cell, which enhances the effect [32]. Apocynin ester (Apo-e, Fig 2.6) is an ester form of apocynin making the molecule more lipophilic to increase oral bioavailability.

Paeonol (PN, Fig 2.7), the main phenolic compound in paeony roots (*Paeonia species*), is used in traditional medicine to treat inflammation [33,34]. Other reported properties of PN are anti-oxidant and apoptosis inducing effects [33].

Pterostilbene (PTS, Fig 2.8) is an anti-oxidant with anti-cancer properties found in berries [35,36]. PTS is an analogue of resveratrol, however with an enhanced oral bioavailability [35].

Pravastatin sodium (PSS, Fig 2.9) is the only included NP of microbial origin. PSS is part of the HMG-CoA reductase inhibitors, generally referred to as statins, which lower low density lipoprotein (LDL) levels [37] and have anti-inflammatory effects in patients with cardiovascular diseases [38].

One of the difficulties in interpreting the relative potency of NPs is the fact that most studies investigate a single molecular species in a single assay. In this benchmarking study, eight NPs are tested simultaneously in four *in vitro* anti-inflammatory assays representing five major pathways of inflammation (Fig 1) and are compared to the reference GC PLP. Inhibition of secretion of pro-inflammatory cytokines TNF-α and IL-6 by macrophages, inhibition of secretion of pro-inflammatory chemokine IL-8 by colon epithelial cells, inhibition of ROS production by polymorphonuclear leukocytes and inhibition of platelet activation. From the collected results, anti-inflammatory profiles were established and compared to PLP to identify potent alternatives.

## Materials and Methods

### Ethical statements

Blood was collected from anonymous healthy human volunteers. All volunteers have provided written informed consent prior to the first donation. The medical ethical board of UMC Utrecht (Utrecht, The Netherlands) and/or Sanquin Blood Supply Foundation (Amsterdam, The Netherlands) provided approval for the experimental protocols.

### Chemicals

Berberine chloride form, Lipopolysaccharides from *Escherichia coli* 055:B5, Penicillin-Streptomycin, Hanks’ Balanced Salts, Zymosan A from *Saccharomyces cerevisia*, luminol, resazurin sodium salt, sulfuric acid, magnesium sulfate, potassium chloride and formaldehyde were purchased from Sigma-Aldrich Chemie BV, The Netherlands. Prednisolone disodium phosphate was obtained from Fagron BV, The Netherlands. Apocynin, apocynin-ester and paeonol were provided by PhytoGenix BV, The Netherlands. Pterostilbene was purchased from Chromadex Inc., USA, pravastatin sodium from AK Scientific Inc, USA and epigallocatechin gallate and curcumin from Chengdu Biopurity Phytochemicals Ltd. Dimethyl sulfoxide, albumin bovine fraction V and 1-step ultra TMB ELISA substrate were obtained from Thermo Scientific BV, The Netherlands. Tween 20 and HEPES were purchased from Acros Organics, Belgium. TNF-α and IL-6 cytosets were acquired from Life Technologies BV, The Netherlands, IL-8 cytoset from R&D Systems, Minneapolis, Minnesota, USA and RPMI-1640 without L-glutamine and L-glutamine 200 mM from PAA Laboratories GmbH, Germany. Fetal bovine serum (FBS) was purchased from Lonza, Belgium, heparine from Leo Pharmaceuticals, Denmark, gelatin from Genfarma, Spain and Percoll from Amersham Pharmacia. Ammonium chloride, potassium bicarbonate and sodium chloride were obtained from Merck BV, The Netherlands. Ethylenediaminetetraacetic acid and sodium bicarbonate were purchased from Baker Inc., USA. TRAP-6 was acquired at Bachem USA. Fibrinogen/FITC polyclonal rabbit anti-human was purchased from DAKO, Denmark and PE mouse/anti-human CD62P from BD Biosciences, USA. Cypridina luciferin analog was obtained from TCI N.V. Europe. TNF-α, IFN-γ and IL-1β were purchased from Immunotools, Friesoythe, Germany. Minimum Essential Medium (MEM), non-essential amino acids and sodium pyruvate were acquired from Gibco, Paisly, Scotland.

### Incubation of RAW 264.7 cells with natural products and PLP

RAW 264.7 macrophages were a gift from the Department of Medical Biochemistry at the Academic Medical Center, Amsterdam, The Netherlands. Cells were cultured in RPMI-1640 medium substituted with 10% FBS, 2 mM penicillin/streptomycin and 2 mM L-glutamine. Cells were split twice a week until maximum passage number 32.

10,000 μM stock solutions of the NPs were prepared one day prior to the assay. For BBCl, a stock solution of 5000 μM was used. All compounds except PLP were first dissolved in DMSO, keeping a DMSO concentration of ≤0.2% for use in the cell culture. Subsequently, the compounds were dissolved to the desired stock concentration using milliQ water.

Cells were seeded into 96-well cell culture plates at a concentration of 5 * 10^5^ cells/mL and left to settle at 37°C in a humidified incubator containing 5% CO_2_. After 6–8 h, the cells were tightly adhered to the bottom of the wells. We have chosen the cell density to allow the cells to reach confluency over the course of the experiment. Less dense seeding of the macrophages resulted in poor and uneven growth of the cell monolayer. The medium was taken off, and concentrations between 4 and 250 μM twice in triplicate per compound were added to the cells and incubated for 2 h in the same incubator. Incubation with medium only was used as a negative control and incubation with PLP served as a positive control. Subsequently, the compound containing medium was taken off and cells were incubated for 12 h with 250 ng/mL LPS to induce an inflammatory response.

To properly establish the incubation times in the RAW 264.7 macrophages, first we established the incubation and stimulation order with different LPS stimulation times. First sample incubation followed by 12 h LPS stimulation proved best for a dose response effect. Preconditioning of the cells to an anti-inflammatory state by the chosen compounds, produces a far more reproducible halt on the LPS stimulatory response, that is in line with the described pharmacological activities of anti-inflammatory compounds. In contrast, LPS stimulation first produces an almost instantaneous activation that is difficult to control at meaningful drug concentrations with a strong dependence of the timing of the intervention.

Only one set of triplicates received LPS, the other set got plain medium instead and served as a negative (unstimulated) control. After LPS stimulation, supernatant was used for cytokine level determination using an ELISA assay (see section 'TNF-α, IL-6 and IL-8 ELISA').

Cells were subsequently checked for their viability using Alamar blue solution (440 μM resazurin salt in PBS). Medium containing Alamar blue (1:10 v/v medium/Alamar blue) was added to the cells and after 3 to 4 h incubation at 37°C, fluorescence was measured at excitation/emission 560/590 nm using a spectrophotometer and compared to untreated cells. The experiment was repeated three times (n = 3).

### Incubation of Caco-2 cells with natural products and PLP

The human colon carcinoma cell line, Caco-2, was obtained from the German Collection of Microorganisms and Cell Cultures (DSMZ ACC 169, Braunschweig, Germany). Caco-2 cells were cultured in MEM, supplemented with 1% (v/v) non-essential amino acids, 1 M sodium pyruvate, 50 U/mL streptomycin, 50 U/mL penicillin and 10% (v/v) heat-inactivated FBS. Cells were grown at 37°C in a humidified incubator containing 5% CO_2_.

For experiments, Caco-2 cells were seeded in 96-wells cell culture plates at 10,000 cells/well and allowed to grow to confluence. After four days, cells were incubated with the compounds at 37°C in a humidified incubator for 2 h. Afterwards, a pro-inflammatory cytokine mix of TNF-α, IFN-γ and IL-1β (final concentrations 10, 5 and 1 ng/mL, respectively) was added and cells were incubated another 16 h at 37°C in the same incubator. After 16 h, supernatant was collected and stored at -80°C for subsequent determination of IL-8 concentrations using a commercial ELISA (R&D Systems, Minneapolis, Minnesota, USA). After aspirating the supernatant, cell viability was checked using Alamar blue (see section 2.2 'Incubation of RAW 264.7 Cells with NPs'). The experiment was repeated three times (n = 3).

### TNF-α, IL-6 and IL-8 ELISA

ELISAs were performed as per suppliers instructions with some minor modifications. Briefly, immuno Maxisorp plates (Nunc art NO. 439454) were coated with coating antibody in PBS, sealed and left to incubate over night at room temperature (RT). Between each step, wells were washed using 0.05% Tween 20 in PBS. After coating, cells were blocked for at least 1.5 h using block buffer (0.5% BSA in PBS for TNF-α and IL-6 and 1% BSA with 0.05% NaN_3_ in PBS for IL-8).

First incubation: wells were either incubated with standards (0–1000 pg/mL for TNF-α and IL-6 and 0–2000 pg/mL for IL-8) in 1:2 serial dilutions in assay diluent (0.5% BSA and 5% FBS in PBS for TNF-α and IL-6 and 0.1% BSA, 0.05% Tween 20 in Tris-buffered Saline for IL-8) in duplicates or with samples (supernatant of incubation assay) in triplicates for 1 h (TNF-α and IL-6) or 2 h (IL-8) at RT in the dark. Second incubation: cells were incubated with detection antibody in block buffer for 1 h (TNF-α and IL-6) or 2 h (IL-8) at RT in the dark. Third incubation: wells were incubated with streptavidin-HRP solution in block buffer for 1 h at RT in the dark.

Plates were developed by adding 1-step ULTRA TMB ELISA substrate and the reaction was stopped after all concentrations of the standard showed coloration, with the same volume of Stop Solution (H_2_SO_4_, 1.8 M for TNF-α and IL-6 and 1 M for IL-8). Optical density was measured at 450 nm using a spectrophotometer.

### Isolation of polymorphonuclear leukocytes from human buffy coats

For the isolation of polymorphonuclear leukocytes (PMNs), human buffy coats (Sanquin Noordwest, Amsterdam, The Netherlands) were poured into 15 mL PBS/heparin solution (10 IE heparin/mL PBS) in a 50 mL tube and mixed carefully. 12 mL Percoll solution (0.15 M NaCl in 1:1.6 Percoll diH_2_O) was put under the buffy without disturbing the forming gradient. Tubes were spun at RT at 1200 *g* for 20 min without break. The top layer containing plasma, mononuclear cells and Percoll was taken off and discarded, leaving the pellet consisting of erythrocytes and PMNs undisturbed. Subsequently, cold lysis buffer (0.16 M NH_4_Cl, 10 mM KHCO_3_ and 0.1 mM Na_2_EDTA in diH_2_O) was added, the tubes swirled carefully and kept on ice until the solution was blackish red. Tubes were spun at 400 *g* for 5 min with break at RT and the supernatant containing the lysed erythrocytes was taken off and discarded. This step was repeated to remove as many erythrocytes as possible. The pellet containing mostly PMNs, was taken up in 10 mL HBSS-gel (4.2 mM NaHCO_3_ in Hank's Buffered Salt Solution (HBSS) substituted with 1% gelatin solution in diH_2_O) and the cell concentration was determined using Türk solution and a hemocytometer. PMNs were diluted until approximately 1 * 10^7^ cells/mL HBSS-gel and were ready to use (see section 'Oxidative Burst Assay').

### Oxidative burst assay

All compounds except PLP were dissolved in DMSO and further diluted using HBSS buffer to the desired stock concentrations. These concentrations were a 4-fold of the highest of the duplicate 1:2 serial dilution series of the compounds: 4000 μM for PLP, PN and PSS, 1000 μM for BBCl, Apo, Apo-e and PTS, 200 μM for EGCG and Cur and 1% for the DMSO control.

EGCG was used as a positive control and HBSS-gel as blank. 50 μL of standards was pipetted in duplicates into white 96-well plates. To this, 50 μL luminol was added as an enhancer of the luminescence and 50 μL PMNs were added. At last, 50 μL human serum opsonized zymosan was added to start the reaction and luminescence was immediately measured for 30 min at 3 min intervals at 37°C (Titertek Luminoskan, TechGen International, Zellik, Belgium). Inhibition of ROS production compared to the blank was measured. The assay was repeated for four different donors (n = 4).

### Platelet activation assay in whole blood

In this assay, PE-anti-P-selectin and FITC-anti-fibrinogen were added to drug stock concentrations made in HBS buffer (10 mM HEPES, 150 mM NaCl, 1 mM MgSO4 and 5 mM KCl) at pH 7.4. Blood from healthy human volunteers was gently mixed with compound solutions at specific concentrations at a ratio of 1:10 (v/v buffer/blood) and were incubated 30 min at RT. Subsequently 60 μM of the platelet activator Thrombin Receptor Activator Peptide 6 (TRAP-6) was added. Fixative solution (154 mM NaCl and 0.2% formaldehyde) was added to samples taken after 1, 10 and 20 min, with a sample to fixative solution ratio of 1:20 (v/v). Fluorescence intensity of P-selectin (PE) and fibrinogen (FITC) was measured by flow cytometry with a FACS Canto II apparatus (BD Biosciences, San Jose, CA, USA) and analyzed with FACSDivaTM software (BD Biosciences, San Jose, CA, USA). 10,000 events were recorded per sample. The assay was repeated for three different donors (n = 3). The area under the curve (AUC) was calculated to determine the extent of platelet activation.

### Scientific calculations

Graphs and calculations were made using GraphPad Prism (GraphPad Prism version 5.0 for Windows, GraphPad Software, Inc [40]). IC_50_s for the assay parameters (*i*.*e*. TNF-α, IL-6, IL-8, ROS and platelets) were calculated using the 'log(inhibitor) vs. response' of the 'non-linear regression of single data' analysis function. The curve was fit following the 'least squares (ordinary) fit', the top of the curve was set to constant equal to 1.0 (to which the data was normalized). Each replicate of Y was considered as an individual point and the curve was fit with a maximum of 1000 iterations and IC_50_s with R^2^ and span (as maximum inhibitory effect, E_max_) were used. For the RAW 264.7 and the Caco-2 cell cultures, data of three separate experiments were used (n = 3), PMNs of four donors (n = 4) and whole blood from three donors (n = 3).

### Molecular structures and property calculation

Chemical structures and CAS registry numbers were resolved using SciFinder [41]. Structures were drawn using MarvinSketch 6.1.5, properties were predicted and calculated using Marvins' Calculater Plugins. Instant JChem 6.1.3 was used for structure database management (all from ChemAxon [39]).

## Results

### Properties of selected compounds

Molecular and structural properties of the selected compounds (Table 1 and Fig 2) reveal that BBCl, Cur, PN and PTS are present in their neutral form at physiological pH as their logP and logD are the same. Only PLP and PSS are negatively charged at physiological pH, the other compounds can only be present in their cationic form at very low pH. Furthermore, the molecular weight (MW) show that all compounds are within the limits of Lipinski's 'rule of 5' [42].

**Table 1:** Identifiers and physicochemical properties of the selected compounds. Full names, their abbreviations as used in the text and CAS registry numbers (CAS RN) are given as compound identifiers. Molecular weight (MW) and log*D* at pH 7.4 were predicted or calculated using Marvins' Calculater Plugins and Instant JChem 6.1.3 was used for structure database management (ChemAxon Kft. [39]). Both the MW and the log*D* of the compounds fit within Lipinski's 'rule of 5' [42].

Name	Abbreviation	CAS RN	MW	Log*D* at pH 7.4
Prednisolone disodium phosphate	PLP	125-02-0	484.39	-2.42
Epigallocatechin gallate	EGCG	989-51-5	458.375	2.97
Berberine chloride	BBCl	633-65-8	371.82	-1.28
Curcumin	Cur	458-37-7	368.39	4.12
Apocynin	Apo	498-02-2	166.18	1.02
Apocynin ester	Apo-e	448251-47-6	222.24	1.68
Paeonol	PN	552-41-0	166.18	1.72
Pterostilbene	PTS	537-42-8	256.30	3.69
Pravastatin sodium	PSS	81131-70-6	446.52	-1.38

### Capacity of the compounds to inhibit the production of pro-inflammatory mediators

The compounds were tested in the following anti-inflammatory assays: inhibition of secretion of pro-inflammatory cytokines TNF-α and IL-6 by macrophages, inhibition of secretion of pro-inflammatory chemokine IL-8 by colon epithelial cells, inhibition of ROS production by polymorphonuclear leukocytes and inhibition of platelet activation (see Fig 1 for these inflammatory pathways).

**Fig 3:**
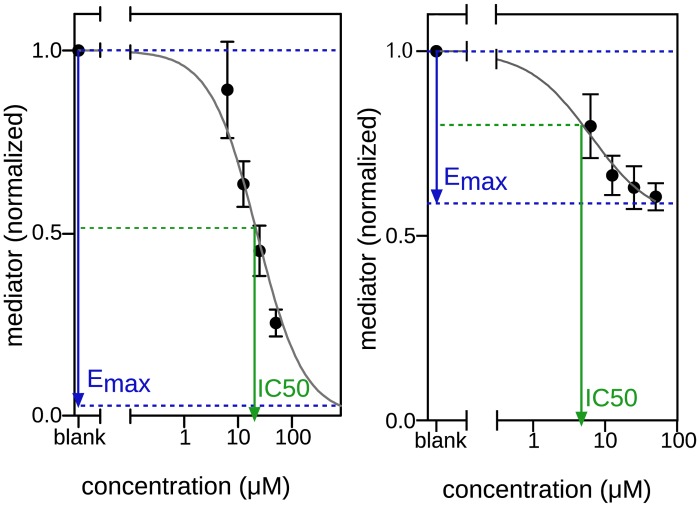
Dose response curve explanation. Two dose response curves are displayed. The first with a maximum effect (E_max_) of close to 100% and the second with an E_max_ of less than 50%. The IC_50_ is the concentration at which 50% inhibition of the E_max_ is observed. Compounds with an E_max_ < 30% and/or R^2^ < 0.70 were considered not to be inhibitory even if an IC_50_ could be calculated.

Sigmoidal dose response curves were constructed as a measure of performance in all *in vitro* assays. For this, the following parameters were used: the maximum effect (E_max_), the concentration at which the compound achieved 50% of the maximum effect (IC_50_) and the curve fit (R^2^). This means that the IC_50_ is not necessarily absolute 50% inhibition (Fig 3). The curve fit is an important measure for the significance of the result; 1.0 means a perfect fit, and 0.1 there is no fit, which means in our case that there is no dose dependent concentration and hence no dose response curve. Compounds which either had an E_max_ of < 30% or an R^2^ of < 0.70 were considered inactive.

### Cell viability

All cells in all four assays were still 100% viable at the highest concentrations used at the end of the incubation period as determined by the Alamar Blue assay. Also, the DMSO concentrations used to dissolve the compounds even for the highest concentrations of NPs were so low (≤0.2% v/v) that it did not interfere with the viability of cells.

### Inhibition of TNF-α and IL-6 secretion by macrophages

Inhibition by LPS stimulated murine RAW 264.7 macrophages of TNF-α and IL-6 secretion was quantified. Inhibition of TNF-α secretion was only observed in cells incubated with PLP and Cur. Although Apo-e has an E_max_ of 30%, the low curve fit (R^2^ = 0.35) makes it a weak and unreliable inhibitor (Table 2). For IL-6 secretion, PLP, BBCl and Cur were inhibitory, whereas the other NPs were ineffective for both cytokines (Table 3). Unstimulated macrophages did not secrete TNF-α and IL-6.

**Table 2:** Inhibition of TNF-α secretion by RAW 264.7 macrophages. PLP and Cur demonstrated an inhibitory effect on TNF-α secretion by LPS stimulated RAW macrophages with IC_50_s of respectively 2.6 and 7.4 μM. The other compounds all had an E_max_ of < 30% and were considered inactive.

Compound	IC_50_ (μM)	E_max_ (%)	Fit (R^2^)
PLP	2.6	45	0.88
EGCG	-	< 30	-
BBCl	-	< 30	-
Cur	7.4	46	0.86
Apo	-	< 30	-
Apo-e	5.2	30	0.35
PN	-	< 30	-
PTS	-	< 30	-
PSS	-	< 30	-

**Table 3:** Inhibition of IL-6 secretion by RAW 264.7 macrophages. PLP, BBCl and Cur demonstrated inhibitory effects on IL-6 secretion in LPS stimulated RAW macrophages with IC_50_s of respectively 3.7, 10.4 and 22.5 μM. The other compounds all had an E_max_ of < 30% and were not considered to be inhibitory.

Compound	IC_50_ (μM)	E_max_ (%)	Fit (R^2^)
PLP	3.7	66	0.97
EGCG	-	< 30	-
BBCl	10.4	44	0.77
Cur	22.5	100	0.84
Apo	-	< 30	-
Apo-e	-	< 30	-
PN	-	< 30	-
PTS	-	< 30	-
PSS	-	< 30	-

### Inhibition of IL-8 secretion by colon epithelial cells

In human Caco-2 epithelial cell cultures stimulated with a pro-inflammatory cytokine mix (TNF-α, IFN-γ and IL-1β) inhibition of IL-8 secretion was measured. Inhibition was only achieved by the NPs EGCG (used as reference), BBCl and Cur. PLP and the remaining NPs did not inhibit cytokine induced IL-8 secretion by Caco-2 cells (Table 4).

**Table 4:** Inhibition of IL-8 secretion by Caco-2 colon epithelial cells. EGCG, BBCl and Cur demonstrated inhibitory effects on IL-8 secretion in pro-inflammatory cytokine stimulated Caco-2 cells with IC_50_s of respectively 78.3, 15.2 and 38.9 μM. The other compounds all had an E_max_ of < 30% and were not considered to be inhibitory.

Compound	IC_50_ (μM)	E_max_ (%)	Fit (R^2^)
PLP	-	< 30	-
EGCG	78.3	100	0.94
BBCl	15.2	52	0.79
Cur	38.9	86	0.76
Apo	-	< 30	-
Apo-e	-	< 30	-
PN	-	< 30	-
PTS	-	< 30	-
PSS	-	< 30	-

### Inhibition of ROS production in human polymorphonuclear leukocytes

ROS production in human PMNs was strongly inhibited by EGCG (used as reference), BBCl, Cur, Apo, Apo-e and PTS. PLP, PN and PSS did not have any inhibitory effect on the production of ROS (Table 5).

**Table 5:** Inhibition of ROS production in human polymorphonuclear leukocytes. ROS production was not inhibited by corticosteroid PLP. Of the NPs, only PN and PSS did not inhibit ROS production. All other tested compounds showed an inhibition of ROS production with E_max_ values close to 100%.

Compound	IC_50_ (μM)	E_max_ (%)	Fit (R^2^)
PLP	-	< 30	-
EGCG	5.9	92	0.91
BBCl	26.5	94	0.99
Cur	7.0	100	0.96
Apo	11.4	95	0.99
Apo-e	14.3	86	0.94
PN	-	< 30	-
PTS	22.2	100	0.83
PSS	> 1000	100	0.53

### Anti-platelet effects in human whole blood

Platelet activation was determined as fibrinogen binding to platelet GP IIb/IIIa integrin receptor and P-selectin expression on the surface of platelets. Platelets were activated with endogenous TRAP-6. Compounds that were able to reduce fibrinogen binding were PLP, EGCG, BBCl and Cur. For P-selectin, PLP, BBCl and Cur were able to reduce the expression. The remaining NPs; EGCG, Apo, Apo-e, PN, PTS and PSS, could not prevent the activation of the platelets (Table 6).

**Table 6:** Inhibition of platelet activation in human whole blood. TRAP-6 induced platelet activation was determined as fibrinogen binding to platelets and PE-selectin expression on platelets. PLP, EGCG, BBCl and Cur inhibited fibrinogen binding with IC_50_s of respectively 22.1, 65.9, 7.7 and 4.6 μM. PLP, BBCl and Cur inhibited PE-selectin expression with IC_50_s of respectively 83.2, 16.5 and 37.2 μM. The other compounds all had an E_max_ of < 30% and were considered to be unable to prevent platelet activation.

	Fibrinogen binding			PE-selecting expression		
Compound	IC_50_ (μM)	E_max_ (%)	Fit (R^2^)	IC_50_ (μM)	E_max_ (%)	Fit (R^2^)
PLP	22.1	35	0.90	83.2	40	0.87
EGCG	65.9	52	0.84	-	< 30	-
BBCl	7.7	64	0.89	16.5	38	0.91
Cur	4.6	60	0.87	37.2	33	0.79
Apo	-	< 30	-	-	< 30	-
Apo-e	-	< 30	-	-	< 30	-
PN	-	< 30	-	-	< 30	-
PTS	-	< 30	-	-	< 30	-
PSS	-	< 30	-	-	< 30	-

### Anti-inflammatory profiles of natural products *in vitro*

**Fig 4:**
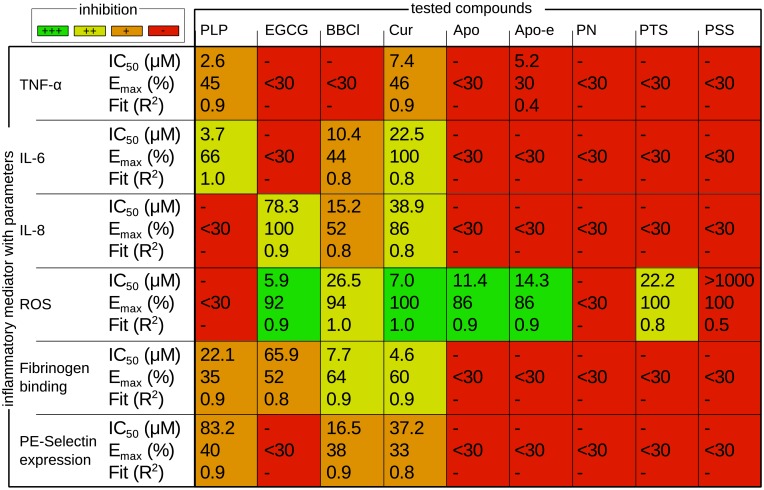
Anti-inflammatory profiles. Performance of the natural compounds compared to the reference corticosteroid PLP is depicted in a colored profile. Cur has an inhibitory effect in all assays, whereas PN and PSS do not induce inhibition in any of the assays. PLP is ineffective to inhibit ROS production, whereas most NPs are very effective. (**strong inhibition, green**) E_max_ > 90%, low IC_50_, R^2^ > 0.70. (**medium inhibition, yellow**) E_max_ > 90%, medium IC_50_, R^2^ > 0.70 or E_max_ > 60%, low IC_50_, R^2^ > 0.70. (**weak inhibition, orange**) E_max_ > 30%, low IC_50_, R^2^ > 0.70. (**no inhibition, red**) E_max_ < 30%, R^2^ < 0.70. PLP = prednisolone disodium phosphate, EGCG = epigallocatechin gallate, BBCl = berberine chloride, Cur = curcumin, Apo = apocynin, Apo-e = apocynin ester, PN = paeonol, PTS = pterostilbene, PSS = pravastatin sodium.

Performance of each of the compounds in every assay was compared in a matrix, providing anti-inflammatory profiles (Fig 4). Different potencies of anti-inflammatory effect were observed based on the determined E_max_, IC_50_ and R^2^ of the dose response curves. Anti-inflammatory potency was classified as follows; strong inhibition (E_max_ > 90%, low IC_50_, R^2^ > 0.70), medium inhibition (E_max_ > 90%, medium IC_50_, R^2^ > 0.70 or E_max_ > 60%, low IC_50_, R^2^ > 0.70), weak inhibition (E_max_ > 30%, low IC_50_, R^2^ > 0.70) and no inhibitory effect (E_max_ < 30%, R^2^ < 0.70).

Cur displayed the broadest anti-inflammatory profile by showing inhibitory activity in all tests. This was followed by BBCl, which was only unable to inhibit TNF-α secretion, but was inhibitory in the other assays. PLP and EGCG have contrasting profiles, only fibrinogen binding to platelets was inhibited to the same extent. Most NPs showed strong ROS production inhibition, whereas PLP was ineffective in ROS production inhibition.

## Discussion

Inflammation is at the foundation of most chronic diseases and thus an important target. GCs are very potent and robust anti-inflammatory agents and widely used to treat inflammation [16], however they possess a broad range of adverse effects [17].

In this study, we have performed an *in vitro* head-to-head performance comparison of eight NPs and prednisolone to identify the most potent NP and best GC alternative. The anti-inflammatory performance of each of the tested NPs has already been described before, however, the approach of simultaneously testing multiple compounds in multiple assays, which is common practice in pharmaceutical research, is novel in phytotherapy research. This way of testing and comparison allows ordering of the (anti-inflammatory) activity and more important the potency of NPs. In order to accomplish this, we have used five key parameters of inflammation, *i*.*e*. inhibition of TNF-α, IL-6 and IL-8 expression, ROS production and platelet activation (as set out in Fig 1). We have chosen for *in vitro* comparison only since the complexity of *in vivo* models is high and makes interpretation of inhibition of multiple inflammatory pathways difficult.

Interestingly, our reference GC PLP, which is one of the most potent anti-inflammatory drugs, does not affect all five pathways. The observed *in vitro* inhibition of TNF-α and IL-6 is consistent with what is described in literature, with prednisolone inhibiting TNF-α secretion by macrophages [43] and lymphocytes [44] and both TNF-α and IL-6 secretion by monocytes [45]. The observed result of PLP to affect platelet function is consistent with what has been described before [46,47]. Interestingly, both IL-8 secretion and ROS production were not inhibited by PLP in our experiments. In literature the inhibition of IL-8 secretion by nasal epithelial cells [48] and inhibition of ROS production in platelets [49] is described, however both studies used different cell lines and stimuli than we used in our assays. The profile of PLP gives us room to select NPs with a similar or contrasting profile, addressing different pathways of inflammation and ideally resulting in less side effects.

Most of the tested NPs, except for PN and PSS, are strong ROS production inhibitors, which is their most important property described in literature [23]. However, for half of them, this is also the only inflammatory pathway they affect and such a narrow profile is unlikely to be competing with that of PLP.

Nonetheless, three of the tested NPs, namely Cur, BBCl and EGCG, do show interesting anti-inflammatory profiles. Cur truly stands out with a stronger and broader anti-inflammatory profile than PLP, inhibiting all tested parameters. This broad profile is consistent with the ability of Cur to combat numerous inflammatory diseases *via* multiple pathways [50]. This makes Cur a promising alternative for GCs like PLP, even the more since PLP is the only GC to inhibit platelet activation so far [47], making the anti-inflammatory profile for GCs as a class even narrower. The broad profile of Cur is followed by BBCl, which is only ineffective in inhibiting TNF-α secretion. Another attractive NP is EGCG, which has a positive performance on the same number of parameters as PLP, addressing the opposite inflammatory pathways except for platelet activation inhibition.

In conclusion, the broader or contrasting profile of the NPs curcumin, berberine and epigallocatechin gallate makes these NPs possible attractive alternatives for GCs like prednisolone disodium phosphate. Because of the poor bioavailability and predominantly hydrophobic nature of these NPs, solubilizers are indispensable. Drug delivery systems are therefore a promising approach to enhance the bioavailability and boost the potency of curcumin, berberine and epigallocatechin gallate as anti-inflammatory compounds.

## Abbreviations

Apo: apocynin
Apo-e: apocynin ester
BBCl: berberine chloride
Cur: curcumin
EGCG: epigallocatechin gallate
GC: glucocorticoid
NP: natural product
PLP: predisolone disodium phosphate
PN: paeonol
PTS: pterostilbene
PSS: pravastatin sodium
